# MiRNA-Based Inspired Approach in Diagnosis of Prostate Cancer

**DOI:** 10.3390/medicina56020094

**Published:** 2020-02-24

**Authors:** Vlad Cristian Munteanu, Raluca Andrada Munteanu, Anca Onaciu, Ioana Berindan-Neagoe, Bogdan Petrut, Ioan Coman

**Affiliations:** 1 Department of Urology, The Oncology Institute “Prof Dr. Ion Chiricuta”, 400015 Cluj-Napoca, Romania; bogdan.petrut@gmail.com; 2 Department of Urology, “Iuliu Hatieganu” University of Medicine and Pharmacy, 400012 Cluj-Napoca, Romania; 3 Medfuture- Research Center for Advanced Medicine “Iuliu Hatieganu” University of Medicine and Pharmacy, 400337 Cluj-Napoca, Romania; muresan.raluca.andrada@gmail.com (R.A.M.); ancaonaciu@gmail.com (A.O.); ioananeagoe29@gmail.com (I.B.-N.); 4 Research Center for Functional Genomics, Biomedicine and Translational Medicine, “Iuliu Hatieganu” University of Medicine and Pharmacy, 400337 Cluj-Napoca, Romania; 5 Department of Functional Genomics and Experimental Pathology, The Oncology Institute “Prof. Dr. Ion Chiricuta”, 400015 Cluj-Napoca, Romania; 6 Department of Urology, Clinical Municipal Hospital, 400139 Cluj-Napoca, Romania

**Keywords:** prostate cancer, biofluids, liquid biopsy, microRNAs, Gleason score

## Abstract

Prostate cancer is one of the most encountered cancer diseases in men worldwide and in consequence it requires the improvement of therapeutic strategies. For the clinical diagnosis, the standard approach is represented by solid biopsy. From a surgical point of view, this technique represents an invasive procedure that may imply several postoperative complications. To overcome these impediments, many trends are focusing on developing liquid biopsy assays and on implementing them in clinical practice. Liquid samples (blood, urine) are rich in analytes, especially in transcriptomic information provided by genetic markers. Additionally, molecular characterization regarding microRNAs content reveals outstanding prospects in understanding cancer progression mechanisms. Moreover, these analytes have great potential for prostate cancer early detection, more accurate prostate cancer staging and also for decision making respecting therapy schemes. However, there are still questionable topics and more research is needed to standardize liquid biopsy-based techniques.

## 1. Introduction

Prostate cancer (PCa) is the most common solid malignancy expanded among men worldwide and it can vary from indolent to very aggressive forms. More than 80% of men with aggressive prostate cancer will develop bone complications, which means a serious decrease in quality of life and survival. At this moment, the diagnostic tools for prostate cancer are digital rectal examination (DRE), prostate-specific antigen (PSA) value, prostate biopsy-Gleason score and prostate magnetic resonance imagining (MRI) [1]. They all conclude with a risk scale that is low, intermediate or high for the failure of treatment at five years. For patients that belong to the high risk group, the five year survival rate is below 30% [2].

PSA screening has led to a decrease in the number of mortality cases, but in spite of this numerical progression, the number of over treatment and over diagnosis cases may have increased. 

After surgery, the continence rate at one year ranges from 85% to 51% for patients undergoing salvage radical prostatectomy [3]. Erectile dysfunctions can occur in up to 65% of cases, varying from poor erections to no erections, especially when nerve sparing techniques are contraindicated or not performed [4].

There is an increasing focus on developing different assays that can evaluate distinct components of body fluids in order to asses more information to complete the whole picture of disease achieved using traditional methods. In this regard, from a molecular point of view, cancer circulating tumor cells, extracellular vesicles, cell-free DNA, circulating RNA and microRNAs can be representative biomarkers in PCa diagnosis and prognosis [5]. 

In this review we discuss the potential of PCa miRNAs associated with tumor growth, progression and metastatic disease. They are valuable, easy to measure biomarkers; they can indicate cancer cell activities and predict the evolution of the disease. MiRNAs can be correlated with PSA values in the circulation, and ISUP Grade Group in order to monitor patients for active surveillance, the decision of treatment or treatment response. In addition, they could lower the unnecessary biopsies, and distinguish indolent cancers from the need to treat cancers [6].

## 2. Current Clinical Point of View for Prostate Cancer Diagnosis

At this moment, the American Urological Association (AUA) and European Association of Urology (EAU) offer guidelines for detecting prostate cancer and the detecting process starts from DRE and PSA values, which lead to prostate biopsy.

It all starts with an elevated PSA cut-off level over 4 ng/mL, or even at a value below that if age-specific PSA is above the level of PSA velocity over 0.35 ng/mL/year. In one of these cases, a prostate biopsy is indicated [7]. At a PSA level of 4 ng/mL with negative digital rectal exam (DRE) findings, the percentage of men detected with positive prostate cancer at biopsy varies from 23% to 38% [8].

Many times DRE can provide false-positive results, which means that the patient will suffer a biopsy with all the complications stated above, or it can provide false-negative results. The DRE sensitivity is 0.51 and specificity 0.59 [9].

TRUS (transrectal ultrasound) is a non-invasive method of assessing the prostate volume, but it is an unreliable tool for detecting prostate cancer [10].

In order to decrease the number of unnecessary biopsies, pre-biopsy information was taken into account such as age, PSA, DRE, prostate volume [11].

**Figure 1:**
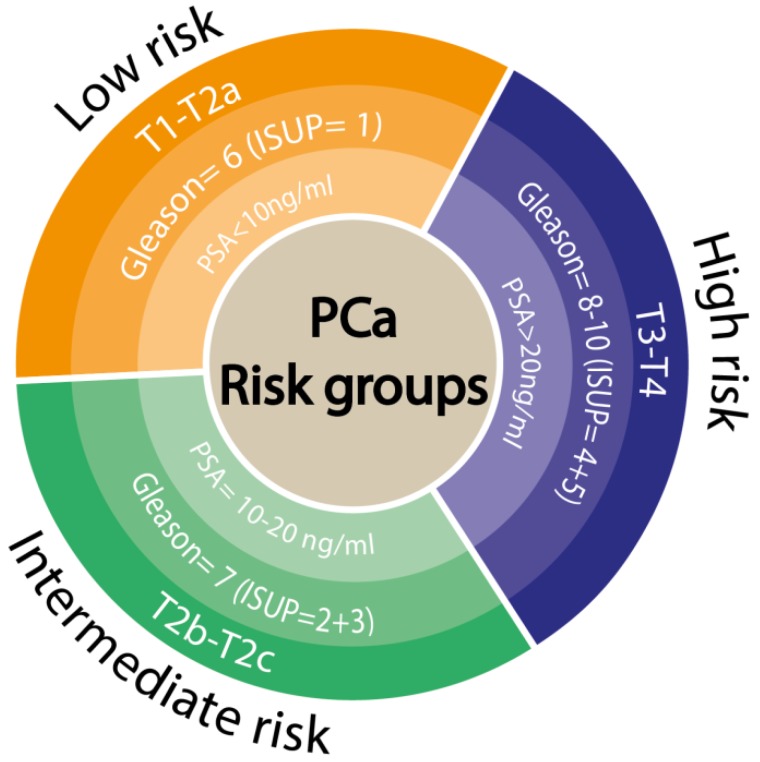
Prostate cancer risk groups classification for localized tumors, referring to biochemical recurrence according to European Association of Urology (EAU) Guidelines (2019).

The pathologist result comes back as a Gleason or ISUP score (Figure 1), which indicates the aggressiveness of the cancer detected. If the tumor is indolent (Gleason 6 or ISUP 1), a type of treatment as active surveillance can be applied. One possible scenario is the upgrading of the Gleason score from biopsy to a radical prostatectomy specimen which means that the aggressive part of the tumor was missed at biopsy [12]. 

In recent years multi-parametric MRI (mpMRI) has emerged as a useful tool to detect suspicious lesions, and the PI-RADS V2 classification is used, with numbering from 1 to 5. This classification is based on morphological suspect lesions. PI-RADS 1 and 2 represent benign tumors, 4 and 5 represent highly suspect lesions for prostate cancer, and 3 is somewhere in between. The higher the Gleason score (ISUP grade), and the higher the tumor volume, the easier it is to detect it. However, there exists a downside; lesions under 1 cm are easy to miss, and even in experienced centers about 16% of cancers can be unnoticed; many are not aggressive, but aggressive and small tumors or multifocal tumors can be missed as well. It is admitted that some clinically significant cancers may be undetected by mpMRI, but in some cases, mpMRI can offer false-positive results [13].

Negative prostate biopsy does not necessarily mean that there is no malignant disease, it actually means that there is no cancer in the specimen examined, and there may be a tumor in the prostate but it was not collected by biopsy.

Complications of prostate biopsy include bacterial infections, hematospermia, hematuria, rectal bleeding, prostatitis or epididymitis urinary retention [8]. Gross hematuria occurs in about 13% of cases, urine retention in about 12%, epididymitis and prostatitis in 5%, UTI 12%, fever and sepsis in about 6.5% each [14]. A negative biopsy can also mean that there is no cancer and the biopsy was made in vain—the patient was subjected to an invasive maneuver without benefits. 

Extensive research has led to the expansion of numerous molecular and genetic assays with promising potential in the development of prostatic cancer biomarkers [15].

## 3. Liquid Biopsy vs. Tissue Biopsy—Transcriptional Point of View

Imagistic results and biopsy present limitations as many other diagnosis instruments, and at this point in the discussion, the next question can be raised: Whether the input of information from this perspective is sufficient to complete the final picture of the diagnosis. The management of prostate cancer patients is often challenging and in the framework of an accurate diagnosis, circulating biomarkers can offer a more specific view regarding clinical problems. 

In the actual context of research in this area, there is extensive research that states that non-coding RNAs like microRNAs represent potential biomarkers for PCa diagnosis. MiRNAs are a class of small RNAs that regulate gene and protein expression [16,17]. Their size varies from 21 to 25 nucleotides and is known to have oncogenic or tumor-suppressive activities. Their level of expression is modified often compared with the healthy specific tissue [18]. Due to the fact that a gene is targeted by multiple miRNAs, they can accelerate abnormal cell growth or induce cell death [19]. Moreover, they can regulate cancer-related events such as epithelial to mesenchymal transition (EMT) and metastasis. They have a role in androgen signaling cascade which is predictive for tumor response to androgen deprivation therapy. 

Tissue biopsy represents the standard method of PCa diagnosis. As mentioned above, this procedure may be performed via a rectal or perineal approach. This is an invasive method, in addition there stand the postoperative complications (haematuria, infection, sepsis), [14] and is directly affected by the competence of the operator. 

On the other hand, liquid biopsies have a non-invasive character and, owing to this property, they lead to significantly less morbidity and can be scheduled at any time in the progression of the surveillance. The principle of liquid biopsy is body fluids collection (blood, saliva, urine) and further analysis of specific biomarkers. This is an approach that is basically done to study circulating tumor cells (CTCs) taking into account circulating DNA, microRNAs and exosomes [20]. Focusing on RNA, Best et al., 2015 evaluated the potential of these molecules by mRNA sequencing of tumor-educated blood platelets, and identified tumors by comparing samples from localized metastasized tumors and healthy patients, with efficiency of 96% [21]. Even if the liquid biopsy presents various patient benefits, it is not yet implemented in current clinical practice, but this aspect may be a matter of time, due to the fact that PSA testing is also a blood-based test that is already a golden standard in the diagnosis of PCa patients.

**Figure 2:**
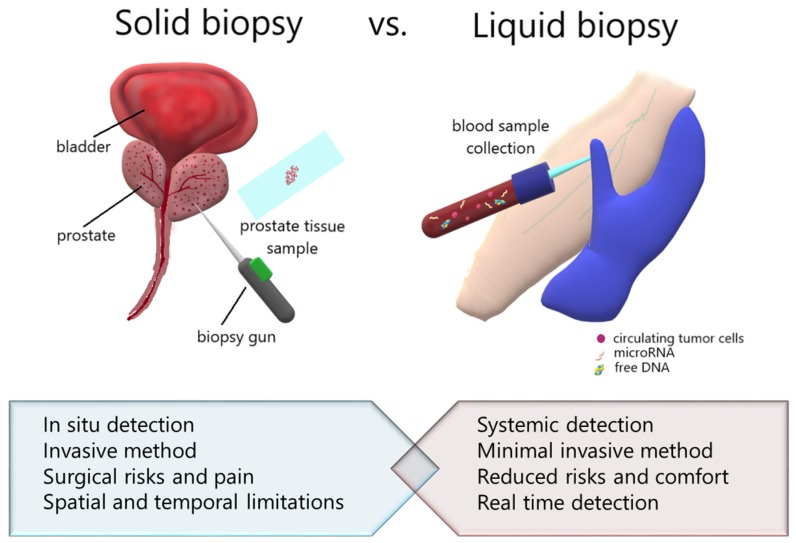
Schematic representation of the principles and main particularities of both procedures used in prostate cancer (PCa) diagnosis.

Figure 2 shows a comparison between solid and liquid biopsy highlighting the principles and properties of these two techniques. On one hand, the most used from a clinical point of view is the solid biopsy, which involves prostate tissue sampling and its anatomopathological analysis. This method is an invasive one and in some cases leads to surgical complications and pain. On the other hand, liquid biopsy may have a lack of risk implication alternatives and is starting to be applied in clinical diagnosis purposes. Biological fluid sampling implies minimal invasiveness and real time detection due to the advanced molecular biology techniques.

This tool of diagnosis has a solid potential to reduce tumor heterogeneity and brings a systemic view of tumor burden [22]. RNA has several advantages to the detriment of DNA, for example, considering the expression at the tissue level; RNA has a higher specificity and has state disease particularities. Secondly, the expression of RNA in cancer cells is a complicated process due to changes that appear during disease development. Last but not least, RNA examination allows the analysis of non-coding RNAs, fusion transcripts and RNA editing events [23,24]. Moreover, mRNA analysis of CTCs can provide information in cases of metastatic castration-resistant prostate cancer. PSA assessment on CTCs comes out as a substitute agent for androgen receptor (AR) signaling, a mechanism that can be explored in androgen therapy decisions [25]. The administration of hormone therapy can lead to reactivation of an AR signaling pathway, and in consequence it drives to a disorder in the functioning of the cells, and this eventually causes castration-resistant terrain. With this background, AR plays a role of transcription factor and regulates genes and ncRNAs. As such, it is anticipated that the long AU-rich 3’-UTR, which is specific to AR, will be targeted by many miRNAs [26].

Moreover, long non-coding RNAs are used also as non-invasive cancer biomarkers, for example, MALAT1 which is the first discovered and had a lot of notoriety in terms of functional and therapeutic agent of interest [27]. MALAT1 appears to be over expressed in bone marrow biopsy samples of castration-resistant prostate cancer [28]. Isolation of CTCs uses a method based on surface markers, for example, EpCAM [29]. The challenge is still present when performing protocols for identifying CTCs because intact cells are discharged into the bloodstream in advanced stages of the disease, and the problem remains the detection of early stages. An interesting result regarding circulating free DNA (cfDNA) was obtained by Reis et al. They demonstrated that the combination of PSA with cfDNA and GADD45a methylation enhances the specificity and the sensitivity in establishing the difference between benign and malignant prostate disease [30].

Further evidence shows that the androgen receptor splice variant, tested positive in CTCs, indicates an aggressive trend in tumor biology development [31].

Scientific research in the field of prostate cancer is showing a growing number of liquid biopsy assays. In the next table are summarized some FDA approved products that are currently used in clinical practice (Table 1).

## 4. MiRNAs Signature in the Diagnosis of Prostate Cancer

Nowadays more than 4800 human mature miRNAs are recorded in miRBase v22 (http://www.mirbase.org). MicroRNAs are considered to be possible bright biomarkers, considering their potential to remain in a stable form in plasma, serum [41] and urine [42]. There is a large spectrum of biological processes regulated by miRNAs with implications in onset, progression and dissemination of cancer.

The association between Gleason score and miRNA transcripts was made by Wang et al. in a study involving 273 miRNA from 62 patients with prostate cancer. This study reported seven strong hot points at miR-16, miR-9, miR-145, miR222, -221, -551a, particularly miR-331-3p, miR-145 are down regulated in higher Gleason scores [43].

The list of miRNAs that are related with prostate cancer is long; for example, Elnaz Pashaei et al. conducted a meta-analysis, including 37 microRNAs, of which 15 over expressed and 22 under expressed in prostate cancer [44]. Of these, only the following microRNAs were correlated with prostate cancer: miR-1 [45,46], miR-133b [47], miR-449a [48], miR-137 [49], miR-370 [50], miR-221 [51], miR-449b [35], miR-125a-5p [52], miR-199a-3p [53], miR-301b [38], miR-340 [39], miR-361 [40], miR-363 [54], miR-98 [55]. MiR-1, miR-133B, miR-449B and miR-221 were reported with significant value in prostate cancer prediction after radical prostatectomy.

In light of the potential biomarker character of miRNAs in PCa diagnosis, from biological fluids, considerable studies have been conducted for the identification of the significant ones (Table 2).

The sensitivity and specificity of some miRNAs listed in the table above present increasing values. For example, the combination of miRs-141, -151-3p and -16 have a sensitivity of 84% and a specificity of 96% regarding mCRPCa detection from liquid samples [59]. Other representative results show that the association of miR-141-3p with miR-21 and miR-375 described a sensitivity of 93% and a specificity of 63% in predicting PCa from serum samples [80].

In the same context of sensitivity and specificity, miR-21 has proved to have a sensitivity of 87.5% and a specificity of 85.7% in terms of clinical stage background [69]. On the other hand, regarding PCa tissue biopsy, it has been reported that miR-21 has an increased sensitivity and specificity around 90% as molecular signature in diagnosis of this pathology [81]. These results taken together with other clinical investigations could guide the therapeutic scheme development. 

In a recent study, Barcelo et al., 2019, reported that a semen exosomes miRNAs investigation could represent powerful tools in prognosis and diagnosis of PCa when correlated with standard PSA tests. The combination between PSA, miR-142-3p, miR-142-5p, miR-223-3p can differentiate PCa patients from BPH patients with an AUC of 0.82. Moreover, the combination between PSA and miR-324-3p, miR-374b-5p has the capacity to distinguish Gleason scores ≥7 [82].

From the literature we extracted the most relevant miRNAs specific to liquid samples (plasma and serum) that can differentiate between healthy and cancer subjects (Table 2). The most significant miRNAs from liquid biopsies were then correlated with their expression from tissue samples by comparing their level in tumor versus healthy adjacent tissue from The Cancer Genome Atlas (TCGA) database. Therefore, the strategy was to identify the miRNAs with similar deregulation in both solid and liquid biopsies in order to determine the potential biomarkers that could be revealed in a minimally invasive manner such as direct evaluation from liquid samples.

**Figure 3:**
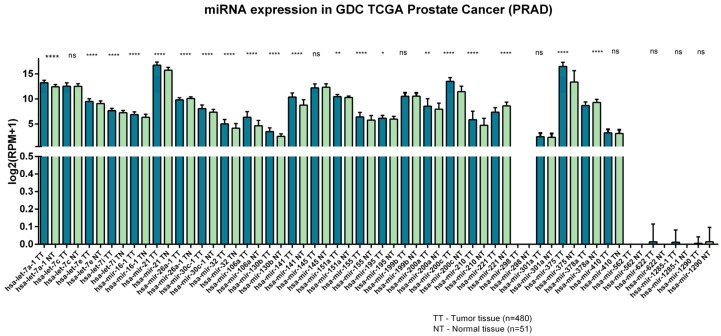
MiRNAs expression level in PCa tissue compared with normal adjacent tissue (The Cancer Genome Atlas (TCGA) database). All bar graphs are displayed as mean ± SEM and the *p*-values were evaluated by unpaired *t* test (ns > 0.05, * *p* < 0.05, ** *p* < 0.01, *** *p* < 0.001, **** *p* < 0.0001).

The “TCGA-PRAD” dataset consists of 480 PCa tumor tissue samples and 51 normal prostate ones. The results show that 18 miRNAs are upregulated and four miRNAs are downregulated in tumor samples versus normal tissue. For example, miR-298 and miR-562 are unexpressed in tissue samples, while miR-622 and miR-1285-1 are found at minimal expression in just a small cohort of cancer tissues and are not expressed in normal ones. MiR-1290 is also found at minimal expression in selective samples from both normal and tumor cohorts (Figure 3). 

MiRNAs found with aberrant expression level in liquid samples from PCa patients were also investigated in terms of their status in tissue samples in order to find the miRNAs that follow the same profile in both types of samples.

**Figure 4:**
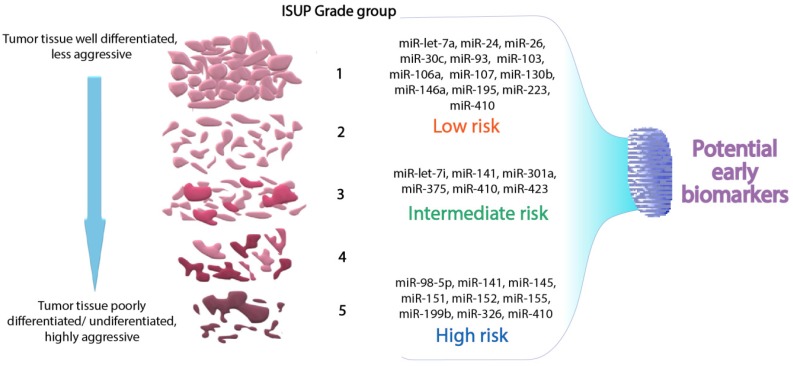
Representation of common miRNAs with value of potential early diagnosis biomarkers, found in solid and liquid biopsy samples according to the TCGA database and the literature.

It is important to mention that miRNA expression levels from data extracted above regarding tissue samples of patients with PCa mostly follow the same trend in liquid samples. In the figure below (Figure 4) the most relevant miRNAs in early diagnosis estimation spread into ISUP Grade categories are represented. 

Moreover, there are miRNAs that are specific for liquid samples like plasma or serum. For example, from these we distinguish miR-4289, which is correlated with high Gleason scores, while miR-103 and miR-451 are correlated with low Gleason scores.

Current revisions have improved Gleason grading and in this frame of references an interesting inverse correlation between miR-125a-3p expression and risk of recurrence was found in human PCa tissues [52].

The MiR-125A-5p up-regulated expression level was associated with prostate cancer cell proliferation and migration. Therefore down-regulation of this miRNA can affect prostate cancer progression [36]. Moreover, miR-125A-5p down-regulation determines NAIF1 gene over expression which also was shown to suppress tumor development [83].

A statement about the correlation between miRNA expression levels and Gleason score affirms that the miR-199a-3p expression level is inversely proportional with the Gleason score and prostate cancer stage. Qu et al., 2014, found that methylation of miR-199a gene is responsible for miR-199a-3p down-regulation in prostate cancer [53]. They also investigated another target of this microRNA, aurora kinase a enzyme, that is directly involved in prostate cancer advancement [84]. Therefore, by targeting miR-199a-3p, aurora kinase A activity will be inhibited [53]. 

Another study of the same groups mentioned above states that miR-199a-3p appears as down-regulated in prostate cancer tissue samples compared with adjacent normal tissue. They also investigated miR-199a-3p status in prostate cancer cell lines where they found the same down-regulated status, which implies cancer cell migration. For this purpose, up-regulation by adding miRNA mimic has a negative impact on cancer cell proliferation and invasion. On the other hand, its direct target, Smad1 over-expression, interferes with the miR-199a-3p anti-proliferative effect on cancer evolution [37].

In a study, E J Noonan et al. determined that miR-449a is often downregulated in prostate cancer pathology, and regulates the proliferation of cancer cells by inhibiting the expression of HDAC-1. This miRNA was not identified in previous studies related to PCa, and microarray studies have shown that this is a promising target with high specificity in this pathology [85]. 

Mortensen et al., 2014, investigated 672 microRNAs and identified 31 linked with prostate cancer biochemical regression after radical prostatectomy. Of these, miR-449B expression was independently related to prostate cancer recurrence in a cohort of 163 patients [35] and can be a representative prognostic biomarker [44]. 

MiR-301a is considered as a diagnostic and prognostic biomarker for prostate cancer patients. MiR-301a expression had presented a higher level in liquid biopsy (serum) and solid biopsy (tumor samples) compared to benign prostate hyperplasia (BPH) patients. This study was performed on two cohorts: The first one composed of 28 prostate cancer patients and 13 controls, the second one composed of 40 radical prostatectomy cases. These results allow an important advancement in prostate cancer prediction due to the possible correlation between miR-301a expression in serum and Gleason score [75] 

MicroRNAs different than the above mentioned are validated as extremely important biomarkers in designing relevant panels for prostate cancer identification especially in early stages. Apart from that, microRNA based research studies are aiming to evolve in the treatment medical area. As a matter of fact, regulating the aberrant expression of microRNAs will inhibit the targets that promote prostate cancer. 

## 5. MiRNAs Therapeutic Role in Prostate Cancer

MiRNAs functional role in PCa disease is not limited only to diagnosis or prognosis biomarkers, these molecules can be recognized also as targets or therapeutic agents. Moreover, there are many studies that explore these agents in the therapeutic field. Due to the fact that miRNAs regulate many target genes, their aberrant expression represents a potential therapeutic value for the normalization of gene expression [86]. 

MiR-130b/miR-301b cluster was found to be up-regulated in prostate cancer. Their oncogenic role was determined by a complex analysis of patient cohort samples correlated with gene expression data [38]. Moreover, miR-301b-3p is an important target since hypoxia, a very common phenomenon [87], will enhance its expression level in prostate cancer [88]. MiR-301b-3p expression level was found to be higher in prostate tumor tissue than in normal adjacent tissue [89]. One of its direct targets, the LRP1B gene, is responsible for low-density lipoprotein receptor-related protein 1B and has a tumor suppressor function [90,91]. For this reason, down-regulation of miR-301b will be a strategic approach for prostate cancer therapy.

MiR-340 has a tumor suppressor role in prostate cancer and can be a potential candidate for further therapeutics development. Furthermore, due to the fact that it is down-regulated in prostate cancer tumor tissue samples and also in cell lines, its up-regulation might be a solution [92]. One of miR-340 actions is to target the 3′-untranslated region of the high-mobility group nucleosome-binding domain 5 and then regulate its expression in order to diminish the cancer pattern. Other proteins’ (cyclin B1, Bcl-2, and matrix metalloproteinase-9) tumorigenic action is influenced by miR-340 over expression [92]. Another target is represented by the MDM2-p53 pathway. In this case, miR-340 affects the 3’ UTR region of the MDM2 protein involved in p53 functional protein regulation, and so cell growth and apoptosis [39]. MDM2-p53 positive phenotype is a significant indicator of prostate cancer aggressiveness [93]. 

A recent study focuses on benign prostatic hyperplasia and its prostate cancer incidence according to miR-340 as a prognostic tool. The research was performed on a cohort of 75 BPH patients and 67 non-BPH patients and revealed that the epithelial to mesenchymal transition is inhibited by targeting the ROCK-1-dependent Wnt/β-catenin pathway in BPH human epithelial cells [94]. 

Respecting the miR-361 family, miR-361-5p was found to be a tumor suppressor marker in prostate cancer. Its direct target STAT6 (signal transducer and activator of transcription-6) influences the over-expression of Bcl-xL (B-cell lymphoma-extra-large), which is responsible for cancer progression [95]. Moreover, it is supposed that androgen-dependent prostate cancer can progress to castration-resistant prostate cancer depending on miR-361-5p down-regulation levels [96]. Another direct target of miR-361-5p is specificity protein 1 (Sp1), a transcription factor associated with the control of metabolism and autophagy, being over expressed in castration-resistant prostate cancer cells. Its inhibition via miR-361-5p over expression will affect cancer malignancy features [40]. 

On the other hand, the androgen receptor is a very important player in this complex scenario because there are other microRNAs involved in the installation of castration-resistant prostate cancer [97].

MiR-363 has shown higher levels in prostate cancer cells compared to normal prostate cells and is considered an oncogenic microRNA. Moreover, in prostate tumor tissue samples, miR-363 expression was found to be up-regulated [98]. Its function respecting cancer cell proliferation mechanisms is controlled by c-myc oncogene which is one of its downstream targets [54]. In addition, pre-miR-363 is involved in miR-106a-363 cluster and targets epithelial to mesenchymal transition transcription factors specific to prostate cancer cells. After radiation therapy, there is an opposite correlation between metastatic features of IFN-induced tetratricopeptide repeat 5 and this cluster expression level [99].

MiR-98 is recognized as a tumor suppressor microRNA controlled by the presence of 1α,25-dihydroxyvitamin D3 [55]. In this regard, miR-98 is a valuable target respecting novel therapies development for prostate cancer. This microRNA is usually found down-regulated in prostate tumor tissues [100]. On the other hand, another member of the miR-98 family, miR-98-5p, was recently recognized as an important candidate in early diagnosis of prostate cancer from plasma samples together with miR-4289, miR-326 and miR-152-3p [76,101]. The encouraging results on miRNAs are becoming palpable rapidly in the context of precision medicine. Recent perspectives indicate that some microARNs may have a dual role, as diagnosis and therapeutic platforms, emphasizing the theranostic area. For instance miRNA-210 was found to be overexpressed in serum of PCa patients, and inhibition therapy generated cell death via apoptosis [102].

## 6. Conclusions and Future Perspectives

In this review, we outlined the compelling research suggesting that circulating miRNAs may serve as prognostic biomarkers in PCa that come as a completion of the actual tools of diagnosis. Moreover, this review should improve the understanding of PCa biology, focusing on the recent research in microRNA transcripts. These transcripts, integrated in the register of liquid biopsies, can provide rapid results with minimal invasiveness that can easily be integrated in clinical practice. Research data based on performing liquid assays demonstrate that this process is feasible and can be done repeatedly at any moment of the surveillance. The collection of data that is generated after the samples are processed is generous, including analytes like RNA, cell-free DNA and CTC with cancer-specific alterations clues.

Taking advantage of liquid biopsies in screenings for detecting early cases is promising, and offers an encouraging possibility to address personalized therapy fast and in real-time. 

Nowadays the research in the medical field is heading for smart device development in order to obtain precise detection and better sensitivity. These intelligent tools are currently tested and most of them use tissue samples from biopsies. Moreover, these tools dispose of artificial intelligence assistance and use various software algorithms aiming for correlations with PCa score categories. In this context, artificial neural networks like convolution ones are widely used for computed guided diagnosis. Such systems were developed by the Prof Dr Hongqian Guo group and other groups [103,104,105].

On the other hand, liquid sample investigation is a promising and interesting alternative for PCa diagnosis because of valuable molecular information that can be discovered. This pathway is somehow more accessible, noninvasive and can facilitate the process of detection. In addition to the mentioned advantages, due to the presence of many analytes (circulating tumor cells, cell-free RNA and DNA and exosomes), liquid biopsy can serve as a prognostic and predictive screening platform for establishing a patient’s treatment too [106]. The molecular profile of these samples provides tremendous significance for clinicians. Transcriptome analysis is the most popular method applied for liquid sample characterization [107].

Of all analytes, the circulating tumor cells present in blood samples are the most studied and have a great impact on PCa classification [108,109].

Nanotechnology and engineering aim to correct the existing gaps in precision medicine by contributing with high throughput perspectives for biological fluid content examination as an indicator of prostate cancer in early stages [110]. Recent progress in liquid biopsy devices describes the use of nanomaterials due to their specific and amazing physical and chemical properties [111]. From this we mention their optical features as plasmonics, which is of considerable interest for SERS (Surface Enhanced Raman Spectroscopy) analysis [112,113]. SERS technique is increasingly employed for prostate cancer detection from body fluids. The most preferred SERS substrates are silver nanoparticles [114], gold nanoparticles targeting different circulating molecules (non-coding RNA, PSA, MUC4, IgG) [115], polymeric membranes [112] and microfluidic devices [116,117].

As a final conclusion, the future investigations regarding the implementation of such novel devices will consider the major benefits for early detection of prostate cancer, among other things. Moreover this molecular level support will serve to create a more complete treatment strategy approach.

## Figures and Tables

**Table 1:** FDA approved liquid biopsy assays suitable for prostate cancer detection.

Product	Methodology	Clinical Evidence	References
Oncotype DX AR-V7 Nucleus Detect	Blood samples are analyzed for the detection of AR-V7 protein in the nucleus that determines the direction of treatment scheme in metastatic castration-resistant cancer patients.	Howard I. Scher et al conducted two studies, one in 2016 and the other one in 2017; with 161 enrolled patients diagnosed with metastatic castration-resistant prostate cancer.	[31,32]
Foundation One Liquid	Next generation sequencing detects clinically relevant genomic alterations like substitutions, insertion/deletions, copy number alterations and selected genetic rearrangements, in 70 oncogenes.	Clark et al. included 860 patients in a study, from which 63 had prostate cancer in 2018.	[33]
Sangia Total PSA Test	A blood sample from fingerstick is collected by the device and analyzed using Claros 1 Analyzer. The test is able to measure total PSA level in the blood in less than 15 minutes.	The cohort was composed of 434 enrolled patients of 50 years old or older from 10 urology clinics from U.S. Sangia Test proved a sensitivity of 84.5% with a 95% confidence interval. The sensitivity increases with almost 59% in conjunction with DRE.	[34]
Progensa PCA 3 Assay	The samples are represented by urine. The test is composed of two quantitative nucleic acid amplification tests that are performed in vitro: One for prostate cancer gene 3 (PCA 3) RNA and the other one for PSA RNA in order to detect the specific amplicons. The assay combines different technologies like transcription mediated amplification and hybridization protection assay in order to quantify the results.	The cohort comprised 495 male subjects enrolled from 14 clinical sites, having a median age of 67 years. From each patient were collected blood, urine and prostatic biopsies. Another study refers to a cohort of 85 patients with prostate cancer or benign hyperplasia. They concluded that DD3 mRNA is another representative biomarker for prostate cancer and its levels can be associated with PSA levels.	[35,36,37,38]
4 K Score Test	Blood samples are analyzed in order to measure four prostate cancer specific kallikreins in the blood: Total PSA, free PSA, intact PSA and human kallikrein 2. The test results undergo some algorithm processing and indicate the aggressivity of prostate cancer prior to prostate biopsy.	Lilja H.’s team included 40,379 men at ages 40, 50 and 60 years, from which they identified 1423 incident prostate cancer patients and 235 with distant metastasis. Zappala S.’s teams conducted two studies. The first study included 1012 men during 2013 and 2014. The second study included 100,000 patients suspected of having prostate cancer. The studies conclude that 4Kscore Test can provide guidance during prostate cancer detection and its results can influence the biopsy decision, therefore reducing the healthcare costs.	[39,40]

**Table 2:** Representative microRNAs found in biological liquid samples of prostate cancer patients that have a promising value in prognosis and diagnosis.

Sample	Cohort	MiRNAs Analyzed Upregulated/Downregulated (↑/↓)	Clinical Value	Ref.
Serum	Seening: 25 mCRPCa, 25 healthy men aged matched Validation set: 21 mCRPCa, 20 healthy men, aged matched	From a total of 365 miRNAs, 5 miRs were studied: miRs-141, -200a, -200c, -210, -375 ↑	Increased levels of circulating miR-, miR-200a and miR-200c are associated with epithelial origin of prostate cancer. miR-210 represents a predictive biomarker. AUC: miR-141: 0.842 miR-200a: 0.638 miR-200c: 0.645 miR-210: 0.652	[20]
Plasma derived circulating microvesicles, serum derived exosomes and microvesicles, urine	Validation set: 21 mCRPCa, 20 healthy men, aged matched	From a total of 742 miRNAs, 12 miRs were selected: miRs-107, -141, -130b, -301a, -2110, -326, -331-3p, -432, -484, -574-3p, -625 ↑ miRs-181a-2*, -572 ↓	In metastatic PCa compared with non-recurrent PCa, miR-375 and miR-141 are significantly increased in plasma exosomes and circulating microvesicles.	[26]
	Serum samples: 47 PCa recurrent patients, 72 non-recurrent patients			
	Urine samples: 17 control patients, 70 local PCa patients, 48 advanced cancer patients			
Serum	25 metastatic PCA patients, 25 matched healthy men	miRs-100, -125b, -141, -143, -205 and -296 were investigated and miR-141 ↑ level was of major importance.	miR-141 AUC 0.907	[41]
Serum	Screening: 7 metastatic PCa, 15 localized PCa Validation set 1: 45PCa Validation set 2: 71 PCa (48N1, 23N0, 29 Gleason >8, 42 Gleason 7), 12 low risk, 12 high risk, 12 intermediate, 12 healthy controls	From 667 miRs, 5 miRs were investigated: miRs-375, -9*, -141, -200b, -516-3p ↑	miRs-141 and -375 are associated with high Gleason score and positive lymph node status.	[56]
Plasma	Validation set 1: 45PCa	From a total of 1146 miRNAs, 5 miRs were selected: miRs-622, -1285 ↑ miRs-let-7e, -let-7c, -30c ↓	miRs- 622, -1285, -let-7e, -let-7c, -30c, combined, differentiate PCa from BPH and healthy men, Area under curve (AUC) 0.924 and 0.860.	[57]
	Validation set 2: 71 PCa (48N1, 23N0, 29 Gleason >8, 42 Gleason 7), 12 low risk, 12 high risk, 12 intermediate, 12 healthy controls			
Serum	28 low risk localized disease, 30 high risk localized disease, 26 metastatic castration resistant PCa (mCRPCa) patients	From a total of 669 miRNAs, 4 miRs were selected. Metastatic castration resistant vs. low risk localized disease: miRs-375, -378, -141 ↑ Low risk vs. metastatic PCa: miR-409-3p ↓	miR-375, miR-141, miR-378 were associated with disease progression.	[58]
Plasma	25 localized PCa, 25 mCRPCa patients	From 742 miRs investigated, 63 miRs were found to be upregulated and 4 miRs downregulated in mCRPCa compared to localized PCa: miRs-141, -375, -200c, -126, -21, -151-3p, -152, -423-3p ↑ miRs-205 and -16 ↓	miR-141, miR-151-3p, miR-16 can differentiate localized and mCRPCa, AUC: 0.944, sensitivity of 84%, specificity of 96%	[59]
Serum	13 BPH, 11 localized PCa, 9 with lymph node or distant metastase (N+/M+), 11 CRPCa patients	From 732 miRNAs studied, 20 miRNAs were selected: miRs-107, -141, -21, -200b, -221, -30c, -346, -375, -574-3p ↑ miRs-1179, -149*, -154, -181a*, -188-5p, -31, -329, -376c, -450a, -508-5p, and -556-5p ↓	mMiRs-let-7a*, -210, and -562 represent promising biomarkers associated with aggressive PCa.	[60]
Plasma exosomes	Screening: 23 CRPCa, androgen deprivation therapy (ADT) failure	miRs-30a/e-5p, -99a-5p, -let-7c, -1246, 1290, -16-5p, -125a-5p, -375 were studied and 2 miRs were representative for the study: miRs-375 and -1290 ↑	High levels of miR-375 and miR-1290 are associated with poor overall survival.	[61]
	Follow up: 100PCa, ADT failure			
Plasma	51PCa (25 metastatic PCa), 20 healthy men	miRs-21, -141 and -221 were analysed and expression level of miRs-221 and -21 was found up-regulated↑.	AUC: miR-21-0.88 miR-221 0.83 did not reach PSA power for discriminating localized from metastatic PCa.	[62]
Serum	18 BPH, 20 healthy men, 37 localised PCa, 8 metastatic	miRs-26a, -32, -195 and -let-7i were studied. PCa vs BPH: miRs-26a, -195 and -let-7i ↑	miR-26a, AUC 0.703, can differentiate PCa from BPH with a sensitivity of 89%, and a specificity of 56%. Combined miRs-26a, -32,-195, -let-7i, PCA vs BPH, AUC 0.758, sensitivity 78%, specificity 67%.	[63]
Plasma	21 PCa patients	miR-141 ↑	Changes of miR-141 are associated with clinical course.	[64]
Serum	25 Healthy men, 25 mCRPCa	miRs-141, -298, -375 ↑ miR-346↓	Expression level of miRs-141, -298 and -375 were increased in PCa.	[65]
Serum	100 treated PCa, N0 (50 low risk, 50 high risk), 50 BPH	From 16 miRs, 12 miRs were detected in serum samples at high levels. miRs-96, -141, 182, 183 were not detectable in >50% of the patients. miRs-let-7a, -24, -26, -30c, -93, -103, -106a, -107, -130b, -146a, -223, -451↑	The investigated miRNAs from serum samples are associated with low grade PCa.	[66]
Whole blood	102 patients: 27 negative biopsy, 75 PCa confirmed	From 12 miRs identified, were selected: miRs-141, -145, -155 ↑ miR-let-7a ↓	Combined miRs-let-7a, -141, -145, -155 AUC 0.783 and PPV of 80%	[67]
Plasma	16 BPH, 59 PCa 11 asymptomatic young men	miRs-let-7c, -30c, -141, -375 were analysed and was noticed that miRs-141 and -375 are down-regulated↓.	AUC 0.809 for miR- 375 Combined miRs-let-7c, -30c, -141, -375 and PSA resulted an AUC 0.877 (sensitivity of 86.8% and specificity of 81.8%).	[68]
Peripheral blood, mononuclear cells	75 healthy men 75 PCa patients	miR-21 ↑	miR-21 AUC 0.9 with a sensitivity of 87.5% and specificity of 85.7%. This miR was associated with clinical stage, tumor differentiation and lymph node metastasis.	[69]
Plasma	36 PCa 31 BPH patients	miRs-1061-5p, -1207-5p, -141-3p, -574-3p, -20a-5p, -21-5p, -93-5p, -2110, -130b-3p, -375 ↑ miRs-223-3p, -24-3p ↓	miR-106a/130b AUC=0.81, miR 106a/223 AUC=0.84, PSA AUC= 0.56.	[70]
Serum	133 patients: 54 PCa 79 BPH	miRs 26a-1 and -141 were investigated and was found an up-regulated ↑ expression level for miR-141	Both miRs failed as diagnostic biomarker, miR-141 levels were increased in high Gleason scores.	[71]
Serum	149 PCa, 81 BPH, 57 healthy controls 40 other urinary pathologies	miR-410-5p ↑	miR-410-5p is a stable biomarker for PCa diagnosis and is associated with low and high-intermediate risk specific Gleason score. PCa vs healthy or other, discrimination, AUC= 0.8097	[72]
Plasma	65 PCa, 51 BPH, 74 healthy controls	miRs-21 and -152 expression levels were analysed and miR-21 has an up-regulated pattern ↑	There was no significant difference in expression between PCa and healthy controls.	[73]
Serum	20 metastatic PCa 31 PCa and 40 healthy patients	miR-141 ↑	Discriminating PCa vs metastatic PCa, AUC 0.8694, PSA AUC 0.7758	[74]
Serum	13 BPH 28 PCa patients	miR-301a ↑	miR-301a expression is correlated with increased Gleason score	[75]
Plasma	Discovery cohort: 42 PCa patients 19 controls	372 cancer associated miRNAs were investigated and 11 miRNAs possible candidates were selected. From this selection: miRs-4289, -326, -152-3p, -98-5p ↑	miRNA panel distinguish between PCa and healthy patients, AUC 0.88	[76]
	Validation cohort: 40 PCa patients 18 controls			
Serum	809 PCa, 241 negative prostate biopsies, 500 patients with other cancers, 41 healthy controls	From 408 miRNAs were selected 38 miRNAs. From these 38 miRNAs, 18 miRNAs were identified as upregulated, from which is important to mention miRs-17-3p and -1185-2-3p, and 2 miRNAs downregulated↓	The combination of miR-17-3p and miR-1185-2-3p achieved a high diagnostic potential with a sensitivity and specificity of 90%, AUC 0.95.	[77]
Plasma	Cohort 1: 98 PCa patients with radical prostatectomy	miRs-182-5p and - 375-3p ↑ in plasma samples	miR-375-3p expression level is a predictor for metastasis development, AUC 0.62	[78]
	Cohort 2: 252 PCa patients before treatment			
	52 healthy donors			
Plasma exosomes	25 localized PCa, 25 with bone metastatic PCa, 10 with pelvic lymph node metastatic PCa patients	An amount of 2588 miRNAs are representative for localized and bone metastatic PCa. 582 miRNAs were differentially expressed in these groups, of which 160 up-regulated and 102 down-regulated. Significant miRNAs: miRs-361-5p, -324-5p, -139-5p, -199b-5p, -199a-3p ↓ miR-632 ↑	Down-regulation of miR-199b-5p is associated with metastatic PCa.	[79]

* MiRNA produced from the opposite arm of the predominant form within the hairpin structure.
